# Lateralization of music processing with noises in the auditory cortex: an fNIRS study

**DOI:** 10.3389/fnbeh.2014.00418

**Published:** 2014-12-09

**Authors:** Hendrik Santosa, Melissa Jiyoun Hong, Keum-Shik Hong

**Affiliations:** ^1^Department of Cogno-Mechatronics Engineering, Pusan National UniversityBusan, South Korea; ^2^Department of Education Policy and Social Analysis, Columbia UniversityNew York, NY, USA; ^3^School of Mechanical Engineering, Pusan National UniversityBusan, South Korea

**Keywords:** functional near-infrared spectroscopy (fNIRS), auditory cortex, lateralization, background noise, music processing

## Abstract

The present study is to determine the effects of background noise on the hemispheric lateralization in music processing by exposing 14 subjects to four different auditory environments: music segments only, noise segments only, music + noise segments, and the entire music interfered by noise segments. The hemodynamic responses in both hemispheres caused by the perception of music in 10 different conditions were measured using functional near-infrared spectroscopy. As a feature to distinguish stimulus-evoked hemodynamics, the difference between the mean and the minimum value of the hemodynamic response for a given stimulus was used. The right-hemispheric lateralization in music processing was about 75% (instead of continuous music, only music segments were heard). If the stimuli were only noises, the lateralization was about 65%. But, if the music was mixed with noises, the right-hemispheric lateralization has increased. Particularly, if the noise was a little bit lower than the music (i.e., music level 10~15%, noise level 10%), the entire subjects showed the right-hemispheric lateralization: This is due to the subjects' effort to hear the music in the presence of noises. However, too much noise has reduced the subjects' discerning efforts.

## Introduction

Asymmetry in functional responses in the right and left hemispheres of a human brain has been observed (Toga and Thompson, 2003). Notable brain asymmetries include the dominance of the auditory cortex in the left hemisphere for speech processing (particularly, the left-side planum temporale region for consonant-vowel syllables, see Jancke et al. (2002), the theory of asymmetric sampling in time (Giraud et al., 2007), and that in the right hemisphere for music processing (Bever and Chiarello, 1974; Tervaniemi and Hugdahl, 2003). This asymmetric response is observed even in infants: speech (Dehaene-Lambertz et al., 2002) and music (Perani et al., 2010). However, Shtyrov et al. (1998) showed that, in a noisy environment, the involvement of the left auditory cortex in discerning speeches considerably decreases, while that of the right hemisphere increases. This reveals that the existence of background noise in spoken words diminishes the dominant role of the left hemisphere in the speech processing (i.e., non-lateralization) (Wong et al., 2008).

Music processing is one of the most complex cognitive activities that the human brain performs. The detailed mechanism of music processing is not well understood yet. Even Bever and Chiarello (1974) had shown that the right lateralization in music processing is even higher among musicians than non-musicians, but still there are contradicting results among different genders, musicians, and non-musicians (Schuppert et al., 2000; Ono et al., 2011). To the best of our knowledge, the influence of background noise in music processing has not been fully investigated yet. The objectives of this study are first to investigate whether, in the presence of noise, the right-lateralization of music processing (in contrast to the speech processing) is destructed or not and second to characterize the noise levels in the music to any differences in the hemodynamic responses between the right and left auditory cortices.

In a live music environment, it will be interesting to categorize the level of noise that would not disturb the music. Under the assumption that the right-hemispheric laterization exists (for some people), it will become the case that the introduced noise does not destroy the right laterization in enjoying the music. Three distinguishing features of a sound are its intensity, frequency, and perception duration, which may elicit different responses to different humans. The study of audition (i.e., hearing a sound transmitted as acoustic waves) involves the examination of the sensory responses to the surrounding environment. To measure sensory responses, various modalities have been pursued in the past: For instance, electroencephalography (EEG) for detecting the language processing (Sinai and Pratt, 2003; Zaehle et al., 2007; Kuhnis et al., 2013b) and the music processing (Meyer et al., 2006; Headley and Pare, 2013) in the auditory cortex, and for detecting the P300 signals in the motor cortex (Turnip et al., 2011; Turnip and Hong, 2012; Khan et al., 2014; Soekadar et al., 2014) magnetoencephalography (MEG) for the auditory cortex (Shtyrov et al., 2012) and the motor cortex (Boulenger et al., 2012), functional magnetic resonance imaging (fMRI) for the auditory cortex (Warrier et al., 2009; Wong et al., 2010) and the frontal cortex (May et al., 2014; Zhou et al., 2014), the positron emission tomography (Tervaniemi et al., 2000), and functional near-infrared spectroscopy (fNIRS) for the motor cortex (Hu et al., 2013) and for the prefrontal cortex (Guhn et al., 2014).

fNIRS is a non-invasive method that measures the absorbed quantity of near-infrared light in the 650~950 nm wavelength range. fNIRS can detect the hemoglobin concentration changes in response to neural activities. For brain imaging, fNIRS offers higher temporal resolution than fMRI and higher spatial resolution than EEG. Particularly, while fNIRS can be used in a natural environment (particularly important in music processing), fMRI should be used in its designated place (or at least a simultaneous hearing and measurement cannot be done without an earphone). Recent fNIRS studies include the hemodynamics analyses in the prefrontal cortex (Hu et al., 2012; Naseer et al., 2013; Santosa et al., 2013; Bhutta et al., 2014), the motor cortex (Aqil et al., 2012a; Kamran and Hong, 2013, 2014), the visual cortex (Hong and Nguyen, 2014), the motor imagery (Naseer and Hong, 2013), and the somatosensory cortex (Hu et al., 2011). Particularly, fNIRS is suitable for studying the auditory cortex because of its non-invasiveness, mobility, cost, and most importantly silence of the equipment. On the other hand, fMRI is relatively problematic in studying the auditory cortex because its measurements are accompanied by acoustic noise resulting from slice selection pulses, cryogen pumping, and magnetic resonance gradient interference (Gaab et al., 2007). To solve the noise problem in fMRI, many schemes have been employed to shield the subject from the machine's acoustic noise, although none of these methods have been proven effective: A few examples are sealing the gradient coil in a vacuum chamber (Katsunuma et al., 2002), using an active noise cancelation (McJury et al., 1997), and utilizing a low-noise gradient-coil design (Mansfield et al., 2001). The noise from an fMRI machine can even interfere with the stimuli designed to evoke neuronal activation: Fuchino et al. (2006) have shown that the level of oxy-hemoglobin in the sensorimotor cortex decreased with the increase of fMRI acoustic noise.

Conducting experiments using fNIRS, on the other hand, is much quieter than using an fMRI system, which makes fNIRS a much more suitable device for experiments related to audio stimulation. Another advantage of fNIRS is that it can detect two main chromophores: oxy-hemoglobin (HbO) and deoxy-hemoglobin (HbR), while fMRI can detect only the BOLD (blood oxygenation level dependent) signal (Plichta et al., 2006). When neuron fires, HbO decreases while HbR increases instantaneously (but it will bring more blood to the area, which will cause the increase of HbO). A drawback of fNIRS is the penetration depth of the light, which is limited to the cortical surface. However, even the relatively poor spatial resolution of fNIRS compared to fMRI (Kovelman et al., 2009) can be ignored due to its other strengths, the relatively faster time resolution than fMRI, low cost, portability, and quietness. While fNIRS enables testing under more relaxed conditions for the subjects, its results on the auditory cortex are relatively rare. The present study is to determine the effects of background noise in music processing: Fourteen healthy subjects participate, and the activations in the auditory cortices are recorded with fNIRS. Specifically, four different sound environments involving ten different conditions are designed for this experiment: music segments only, noise segments only, music segments including noise, and the entire music with noise segments. To investigate this, we examined the hemodynamic responses from 44 channels for three noise categories in both hemispheres.

Our interest exists in finding the (subjective) level of noise that does not disturb listening to music as well as hemispheric lateralization. The following questions will be pursued: (i) Are there more hemodynamic changes in the right auditory cortex than the left auditory cortex, when people hear music. Instead of listening to continuous music, music segments will be exposed to the subjects so that they can focus on. (ii) Can noise alone cause a similar behavior like music segments, or will it bring any difference? (iii) What would be the level of noise that distort the hearing status? (iv) What would be the noise enterance effects when listening to the entire music. As a feature to tell any difference, the gap between the mean value and the minimum value of the hemodynamic responses caused by various conditions will be used.

## Materials and methods

### Subjects

Fourteen subjects (age: 28 ± 5 years; 7 males and 7 females, 12 right-handed and 2 left-handed) participated in the experiment, see Table 1. In this study, the handedness was obtained by asking the subjects about a better performance for use of a hand. Thirteen subjects knew (understood) the music and three subjects were musicians. The definition of musician in this work is whether they are able to play the music with piano or not. The response between musicians and non-musicians is known distinctly different (Kraus and Chandrasekaran, 2010; Kuhnis et al., 2013a, 2014). All musicians (1 male and 2 females; primary musical instrument: piano; mean age 25 ± 2 years) started their musical training between 6 and 12 years. They had more than 16 ± 2 years of musical training and practiced their musical instrument for 1.5 h/day when they started to learn piano. We selected the same number of subjects between male and female to examine a possible gender difference in the hemispheric lateralization (Shirao et al., 2005). All of them had normal hearing and none had a history of any neurological disorder. To reduce noise and artifacts, the subjects were asked to remain relaxed with closed eyes by enjoying the music and to avoid motions during the experiment (i.e., head movement, eye blinking, etc.). During the experiment, the subjects were asked to listen the auditory stimuli attentively (not passively), since selective attention is important to the activation pattern in the auditory cortex (Jancke et al., 1999). Moreover, musicians were asked to imagine playing the music with a piano. The work was approved by the Institutional Review Board of Pusan National University. The subjects were informed about the experimentation and written consents were obtained, which was conducted in accordance with the ethical standards encoded in the latest Declaration of Helsinki.

**Table 1:** **14 Subjects in experiment**.

**Subject**	**Gender**	**Age**	**-handed**	**(Pre-knowledge on the music)**
1	Male	29	Left	Yes
2	Male	27	Left	Yes
3	Male	34	Right	No
4	Male	29	Right	Yes
5^*^	Male	26	Right	Yes
6	Male	31	Right	Yes
7	Male	30	Right	Yes
8	Female	26	Right	Yes
9	Female	25	Right	Yes
10	Female	23	Right	Yes
11	Female	28	Right	Yes
12^*^	Female	25	Right	Yes
13^*^	Female	23	Right	Yes
14	Female	26	Right	Yes

* *They can play the music*.

### Audio stimuli

**Figure 1:**
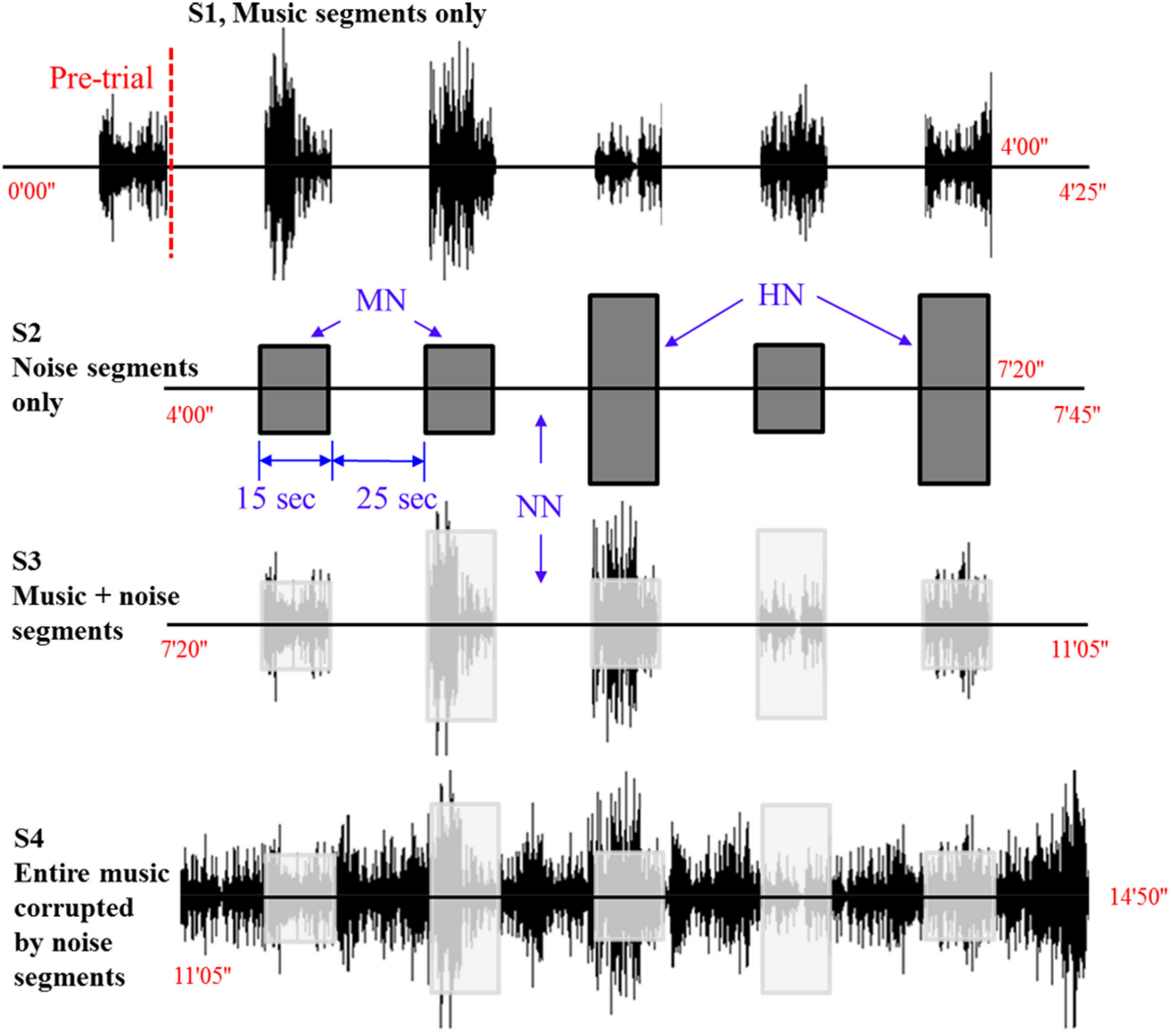
**Experimental paradigm**. Four different sound conditions. The level of noise (i.e., NN, no noise; MN, mid-level noise; HN, high-level noise) changes in a pseudo-random order.

Figure 1 shows the experimental paradigm used in this work. The audio stimuli (music) was *Für Elise* composed by Ludwig van Beethoven. The entire length of the music was 165 s. One experiment consists of four stages involving ten conditions. The first 200 s after the pre-initiation trial constitutes Stage 1 (S1), which examines the basic condition (that is, a music hearing). In S1, the subjects are exposed to 25 s rest and 15 s music for 5 times (it is noted that the pre-initiation trial is not included in the analysis). The objective of S1 is to examine which side (right or left) in the brain is more active upon a musical stimulus. Stage 2 (S2) is the next 200 s after S1 that involves two different noise levels. Therefore, S2 constitutes three conditions: no noise (NN), mid-level noise (MN), and high-level noise (HN). The objective of S2 is to examine whether the noise alone can cause a similar response like the music in S1. Then, the subsequent 200 s after S2 becomes Stage 3 (S3), in which mixed music and noise segments are repeated 5 times. The objective of S3 is to find out whether a music + noise segment will induce more efforts in the brain than the cases of music or noise segments alone. Distinguishing NN, MN, and HN, 3 conditions are examined in S3. Finally, the interruption of noise when hearing the music has been mimicked in Stage 4 (S4). For comparison purposes, similar noise conditions like S2 and S3 are made in S4, yielding another 3 conditions. The noises introduced to the music appear in a pseudo random order. The durations of music and noise segments in S1~S4 are the same. The shaded boxes in S3 and S4 indicate the time periods where noises enter the music. The total experimental time was 14 min and 50 s. The music and noises were digitally mixed using the Adobe Audition software (a WAV-format file: 16 bit, 44,100 Hz, stereo). The same earphone (Sony MDR-NC100D; digital noise canceling earbuds) with the same sound level setting was used across the subjects.

### Noise levels

The *y*-axis in Figure 1 denotes the relative amplitude of the audio signal (its max. scale has 30,000 sample values, which corresponds to 100%) in the Adobe Audition software. In this work, the high-level noise is defined to be the white noise whose amplitude reaches 20% of the full scale. The mid-level noise means 10% in the full scale. To provide variability, the noises were introduced in a pseudo-random order (15 s for each stimulus). The average amplitude of the music was about 10–15% in the *y*-axis.

### fNIRS data and processing

**Figure 2:**
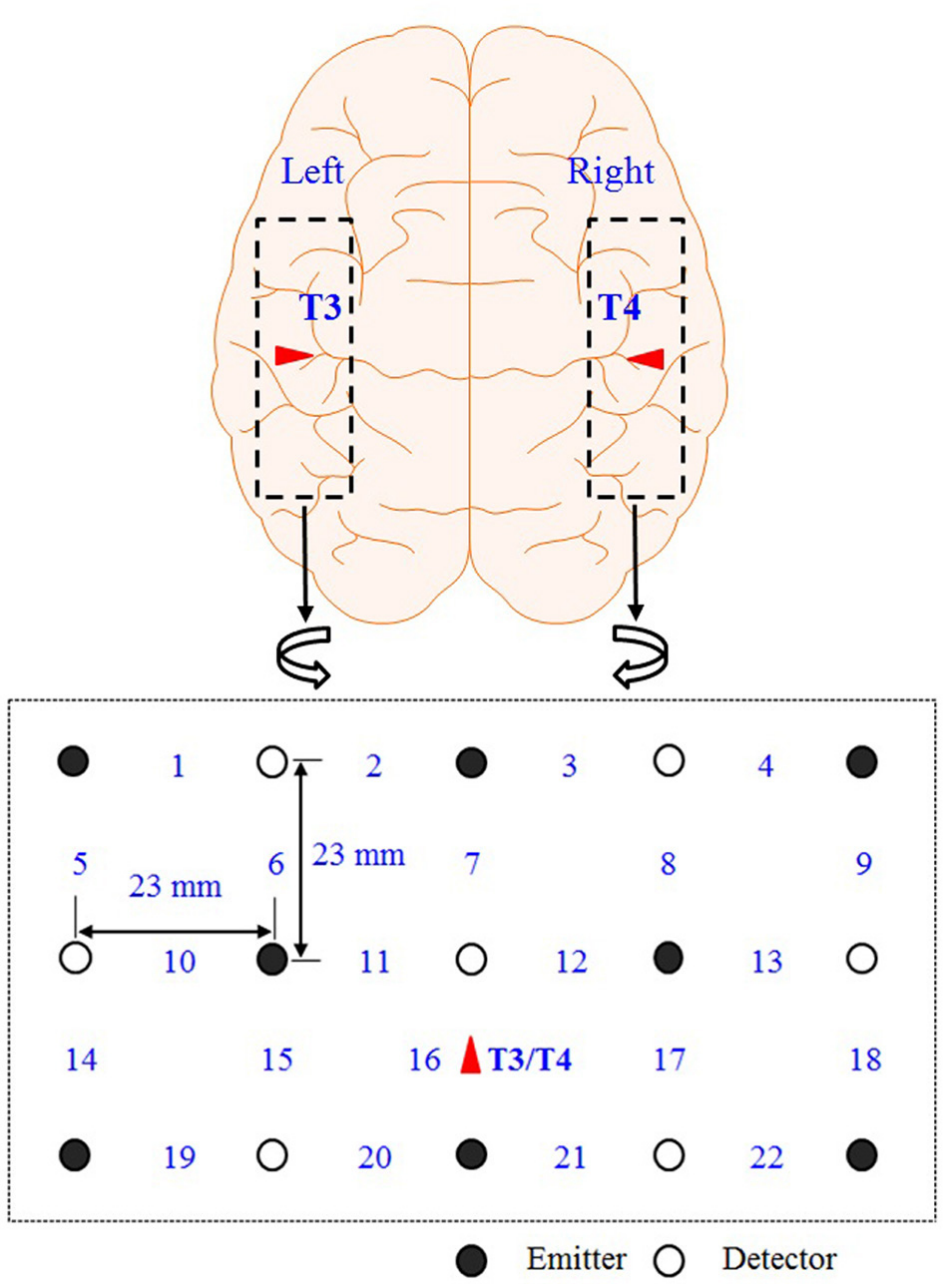
**Optodes configuration**. Numbers represent the measurement channels: The channel number 16 coincides with the T3/T4 locations in the International 10–20 System.

Figure 2 shows the optodes configuration of the fNIRS system (DYNOT: DYnamic Near-infrared Optical Tomography; NIRx Medical Technologies, Brooklyn, NY) for imaging the auditory cortices in the left and right hemispheres. The distance between an emitter and a detector is 23 mm. The data were acquired at a sampling rate of 1.81 Hz and for two wavelengths (760 and 830 nm). A total of 22 channels were measured from 8 emitters (black circles) and 7 detectors (white circles) in both hemispheres. All the lights in the room were turned off during the experiment to minimize signal contamination from the ambient light sources. The optodes were placed on the scalp above the left and right auditory cortices. Ch. 16 in both left/right hemispheres was set to coincide with the T3/T4 locations, respectively, in the International 10–20 system. Since the optodes configuration in Figure 2 covers the entire auditory cortex, the averaged value from the 22 channels is used in the analysis.

Relative changes of the concentrations of HbO and HbR were computed using the modified Beer-Lambert Law as follows,

(1) $$\Delta A_{i}(\lambda ,k)=\left[a^{\text{HbO}}(\lambda )\Delta c_{i}^{\text{HbO}}(k)+a^{\text{HbR}}(\lambda )\Delta c_{i}^{\text{HbR}}(k)\right]\;d_{i}l_{i},$$

where Δ*A*(·, *k*) is the absorbance variation at time *k*; the subscript *i* denotes the channel number; λ is the wavelength of the laser source; *a*^HbO^ and *a*^HbR^ are the absorption coefficients of HbO and HbR, respectively; Δ*c*^HbO^ and Δ*c*^HbR^ are the concentration changes of HbO and HbR, respectively; *d* is the differential path length factor (in this study, constant values *d* = 7.15 for λ = 760 nm and *d* = 5.98 for λ = 830 nm were used for all the channels), and *l* is the distance between a source and a detector.

To analyse the fNIRS data, the open-source software NIRS-SPM (Ye et al., 2009) was utilized in our own Matlab® (Math-works, Natick, MA) code. The respiration and cardiac noises contained in the hemodynamic responses were removed by a low-pass filter of a cut-off frequency 0.15 Hz.

### Classification feature

**Figure 3:**
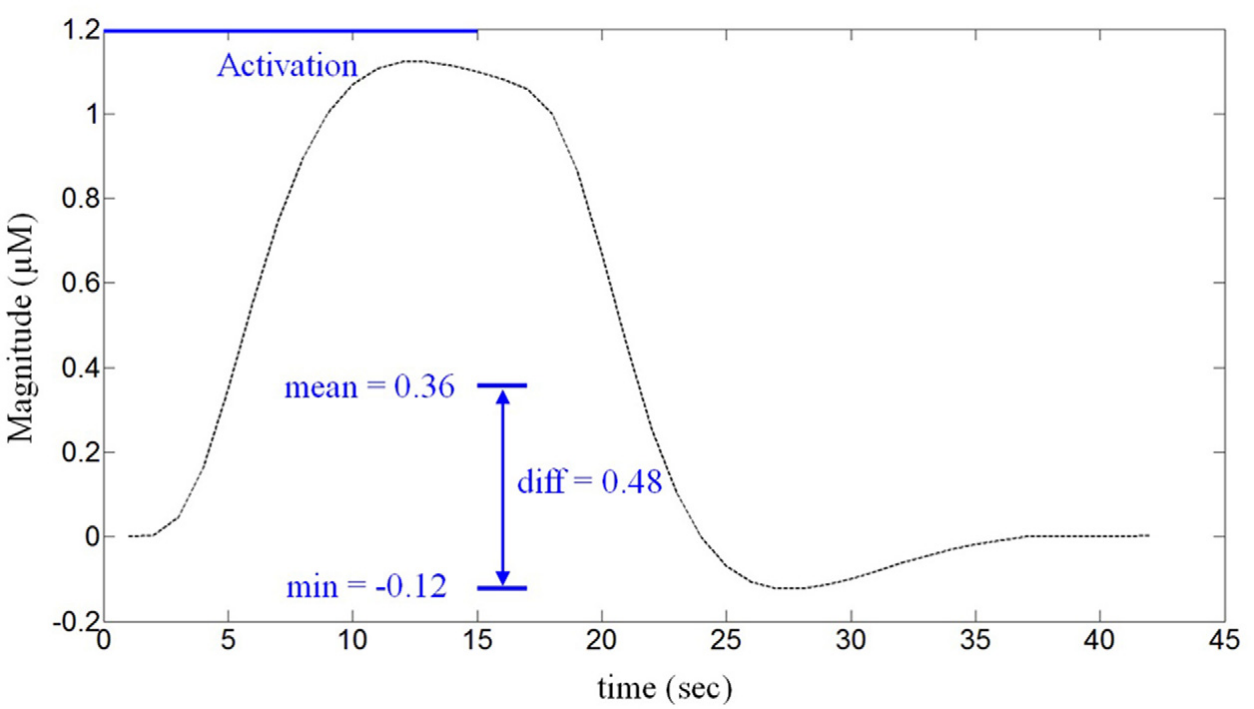
**The defined feature for classification**. The difference between the mean and the minimum value of the HbO obtained from a 15 s stimulus (in μM).

The difference between the mean and the minimum value of a given hemodynamic response has been used as a feature to classify whether the hemispheric lateralization occurred or not. Figure 3 depicts a typical activated hemodynamic response obtained by convolving a stimulus pattern and the impulse hemodynamic response function as

(2) $$h_{\text{M}}\text{​}\left(k\right)=\sum_{n=-\infty }^{\infty }Box\text{​}\left(k\right)h\text{​}\left(k-n\right)\text{​},$$

where *h*_M_ (*k*) denotes the “modeled” hemodynamic response to be used as a reference signal, *Box*(*k*) is the box-type stimulus pattern (in this paper, the 15 s activation period) and *h*(*k*) is the impulse hemodynamic response function adopted from the SPM8 (Wellcome Trust Centre for Neuroimaging, London, UK) (Friston et al., 2008). For example, the curve in Figure 3 shows that the mean value is 0.36 μM, the minimum value is -0.12 μM, and therefore the difference is 0.48 μM. The reason for using the gap between the mean and the minimum value of the hemodynamic response as a classification index in this work is that, with the current experimental paradigm in Figure 1, the hemodynamic response may not come back to the baseline value in a short time interval after each stimulus. Actually, it was so and the baseline drifting has been compensated by the mean-min difference.

### Statistical analysis

The estimation of the cortical activation is the most important factor in the analysis of fNIRS data. Previous studies showed that the activation could be statistically estimated by fitting the measured response to a regression model (Hu et al., 2010; Aqil et al., 2012b). Let **h***^j^_i_* ϵ *R*^*N* × 1^ be the measured fNIRS data at the *i*-th channel, the superscript *j* denote the *j*-th stimulus (i.e., the total 20 stimuli obtained by 5 stimuli/condition × 4 conditions), and *N* be the size of fNIRS data for each stimulus (in this study, *N* = 72 for the period of 15 s stimulus and 25 s rest using the sampling frequency of 1.81 Hz). Then, the general linear regression model is formulated as follows,

(3) $$h_{i}^{j}=\phi_{i}^{j}h_{\text{M}}+\psi_{i}^{j}\cdot 1+\varepsilon_{i}^{j},$$

where **h**_M_ ϵ *R*^*N* × 1^ is the modeled hemodynamic response obtained by (2), **1**ϵ *R*^*N* × 1^ is a column vector of ones for correcting the baseline, ϕ is the unknown coefficient that indicates the activity strength of the modeled hemodynamic response, ψ is the coefficient to compensate the baseline drift of the signal, and ε ϵ *R*^*N* × 1^ is the error term in the regression model. In this paper, the coefficient ϕ is estimated by using the *robustfit* function available in Matlab™ as follows,

(4) $$\left(\left[\begin{array}{c} {\hat{\phi }}_{i}^{j}\\ {\hat{\psi }}_{i}^{j} \end{array}\right],stats\right)=robustfit([1\;h_{\text{M}}],h_{i}^{j}),$$

where ${\hat{\phi }}_{i}^{j}$ and ${\hat{\psi }}_{i}^{j}$ denotes the estimate of ϕ*^j^_i_* and ψ*^j^_i_*, respectively, and *stats* denotes the statistical data including the *t*-value, *p*-value, standard errors, etc.

The idea is to test the null hypothesis that the estimated parameter ${\hat{\phi }}_{i}^{j}$ is equal to zero or not. Furthermore, if ${\hat{\phi }}_{i}^{j}$ is positive, the particular activation is assumed to be active, and if it is negative, the particular activation is not active for the *j*-the stimulus at the *i*-th channel, in which the *t*-value test has been used. In this paper, the *t*-value was computed using the following equation,

(5) $$t_{i}^{j}\;=\;\frac{{\hat{\phi }}_{i}^{j}}{SE({\hat{\phi }}_{i}^{j})},$$

where *SE* is the standard error of the estimated coefficient. We used two criteria to assess the selection reliability of a particular activation for further analysis. They were *t^j^_i_* > 0 and *p^j^_i_* <α, where *p* denotes the *p*-value (in this work, α = 0.05 was set). Alternatively, it could be done by checking *t^j^_i_* > *t*_crt_, where *t*_crt_ denotes the critical *t*-value that depends on the degree of freedom (i.e., 71, which is *N* -1). In this case, *t*_crt_ = 1.994.

## Results

The hemodynamics changes in the auditory cortex upon the occurrence of noises in music processing have been examined. Using the paradigm in Figure 1, four different stages involving ten conditions in combination with three different noise levels were tested. The three-digit numbers in Table 2 indicate the differences between the mean and the minimum value of the HbO concentration change in both left and right auditory cortices for 14 subjects (Subject 1~14). S1~S4 represent four different experimental stages in Figure 1. It is reminded that only those hemodynamic responses whose *p* < 0.05 and *t* > 0 were counted for averaging over 22 channels. It is also noted that (i) the data outside the 3 standard deviations were excluded (i.e., 9 cases in bold-italic in Table 2 were outliers, see Subject 5 and Subject 12) and (ii) if a right-left difference is not significant (i.e., difference < 0.1σ), it was considered to be the same (they were marked in italic font). Table 3 summarizes Table 2. The following observations are made:

- Music segments stage (S1): This stage was to determine which brain side was dominant in music processing. Nine subjects showed the higher activation in the right auditory cortex, whereas three subjects showed it in the left auditory cortex (out of 14 subjects, Subject 12 was an outlier and the data of Subject 10 was undistinguishable). Therefore, the right-hemispheric lateralization in music processing is about 75% (i.e., 9/12).
- Noise segments stage (S2): This stage intended to characterize the auditory reponses to various noises. When there was no noise (see the first row in S2), a significant difference was not seen between the right and left auditory cortices, considering that two data (Subject 10, Subject 14) were un-distinguishable and one data (Subject 12) was an outlier. But, in the mid- and high-noise conditions, there was a slight increase in the number of subjects who showed the higher hemodynamics response in the right auditory cortex (i.e., 15/23, which is about 65%).
- Mixed music and noise stage (S3): Contrary to S1 (music only) and S2 (noise only), mixed signals of noise and music were exposed to the subjects. Considering that the sound level of the music was about 10~15%, the mid-level noise and music segments showed the highest level of right-lateralization over the subjects (see the second row in S3). Actually, removing two undistinguishable subjects (Subject 11, Subject 14), all twelve subjects out of 14 subjects showed the higher activation in the right auditory cortex. This is due to that the subjects tried to hear the music in the presence of noise (recall that the noise level was 10%). But, if the noise level became higher than the sound level of music (i.e., the high-noise condition, 20%), the number of subjects who showed the higher activation in the right auditory cortex had reduced to 10. Another interesting observation was found from the first row in S3, which provided no sound in the middle of music + noise segments. No sound itself had shown almost the same level of right-lateralization in terms of number of subjects as the case of music + high-noise condition.
- The entire music with noise segments (S4): The final stage was to investigate the effect of noise interruptions in hearing music. When there was no noise (see the first row in S4), the tendency of the right-hemispheric lateralization was exactly the same as the case of S1 (i.e., 75%). Also, even upon the interruptions of noise, the tendency was similar.

**Table 2:**
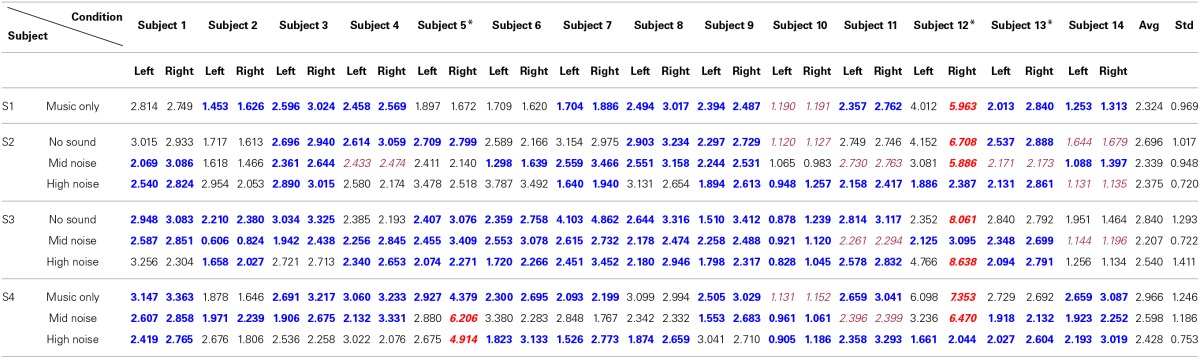
**Differences between the mean and the minimum value of the HbO concentration (unit: μM, scale-up by 10^−4^)**.

*Subs. 1~7 are males and 8~14 are females. Bold-face numbers indicate that the mean-min difference in the right hemisphere is larger than that in the left hemisphere*.

^*^indicates the subjects who can play the music.

**Table 3:** **Statisics of hemispheric lateralization (from Table 2)**.

		**Left is higher**	**Undistinguishable (diff. < 0.1 Std)**	**Right is higher**	**Outliers**
S1	Music only	3(0)	1(1)	9(5)	1(1)
S2	No sound	5(1)	2(2)	6(3)	1(1)
	Mid noise	3(1)	3(2)	7(3)	1(1)
	High noise	5(1)	1(1)	8(5)	0(0)
S3	No sound	3(2)	0(0)	10(4)	1(1)
	Mid noise	0(0)	2(2)	12(5)	0(0)
	High noise	3(1)	0(0)	10(5)	1(1)
S4	Music only	3(2)	1(1)	9(3)	1(1)
	Mid noise	3(1)	1(1)	8(4)	2(1)
	High noise	4(1)	0(0)	9(6)	1(0)
	Grand total	32(10)	11(10)	88(43)	9(7)

*The numbers in the parenthesis indicate the number of females (overall: 73.3%, female: 81.1%, male: 67.2%)*.

For the 140 cases (i.e., 14 subjects × 10 conditions), 9 cases were outliers, 11 cases showed no significant difference, 32 cases showed the higher left auditory cortex, and finally 88 cases showed the higher right auditory cortex. Therefore, the overall lateralization across all the experimental conditions was about 73.3% (i.e., 88/120); specifically, for female 81.1% (i.e., 43/53) and for male 67.2% (i.e., 45/67). It is also noted that 20 cases were excluded.

Now, for analyzing the tendency/strength of lateralization, only the cases showing that the right hemisphere is more active than the left hemisphere were further considered (bold font). Table 4 shows the average and its standard deviation of such cases. That is, the value 2.080 in S1 is the average of 1.453, 2.596, 2.458, 1.704, 2.494, 2.394, 2.357, 2.013, and 1.253 from Table 2. The following observations are made. (i) Comparing S1 and S4 in Table 4, the background noise intensifies the hemispheric lateralization (i.e., the Right Avg—Left Avg value has increased from 0.311 for music only condition to 0.533 for the mid-noise condition and 0.743 for the high-noise condition, respectively). (ii) As seen in the last column of S2, the difference in the mid-noise condition (i.e., 0.536) is larger than the other two cases (i.e., 0.315 and 0.403). This may reveal the fact that too much noise does destroy the subject's discerning efforts for music in the presence of noise. (iii) In S3, even if there is no sound, the difference in the right auditory cortex was higher than that of the left (i.e., 0.566). This reflects that, in the presence of noise, the subjects tried to listen to the music and this effort was continued even if there is no sound. A similar effort from the subjects was also shown in the mid- and high-noise conditions. Overall, the discerning efforts for music from noises has been seen, which is consistent throughout all three conditions.

**Table 4:**
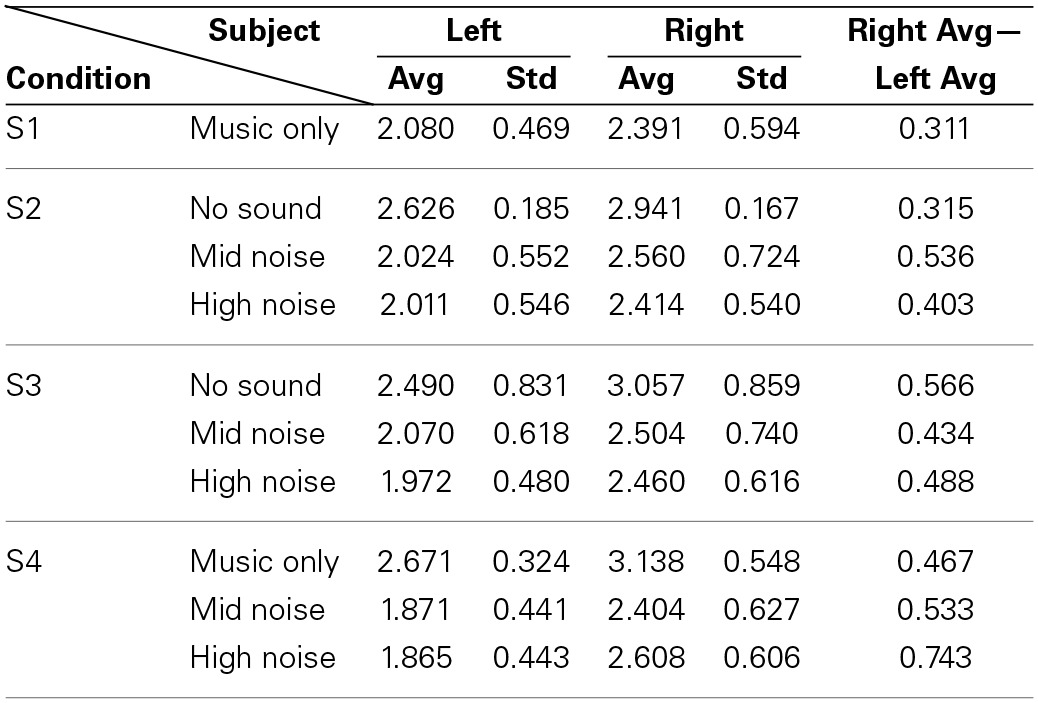
**Average of only those cases where Right > Left from Table 2**.

*Avg and Std denote average and standard deviation, respectively*.

## Discussion

This was the first fNIRS study to examine whether auditory-cortex activation by background-noise and music stimuli could change the hemispheric lateralization in both hemispheres. To investigate the effects of noise on music processing, the participants were subjected to various levels of noises in different conditions in the experiment. It was found that exposure to any noise compared to no noise led to higher auditory-cortex activation in the right hemisphere. This result, in fact, is in line with the previous MEG data on speech stimuli (Shtyrov et al., 1998), wherein instead the left hemisphere was dominant in the perception and production of speech. However, when high-level noise was introduced, activation was observed in both hemispheres resulting in a lack of lateralization in auditory-cortex activation. This clearly demonstrates that auditory-cortex activation by music stimuli with noise changes the hemispheric lateralization when a sufficiently loud distraction is introduced to reduce the specialized function of the brain. Similar results from the previous studies on speech stimuli show that lateralization occurs in different responses for noisy condition.

fNIRS have its advantages over fMRI. Previous studies have employed fMRI to determine the effects of various stimuli on neuronal activation in the auditory cortex (Scarff et al., 2004). The drawback of this modality is the problematic effect of acoustic noise from baseline activity in the auditory cortex. Whereas fMRI can measure the changes in magnetic susceptibility in the blood, fNIRS can directly measure hemodynamic changes, HbO and HbR. fMRI signals are physiologically ambiguous, because they reflect changes in cerebral blood flow, cerebral blood volume, and oxidative metabolism. Many studies have investigated the correlation between fMRI and fNIRS signals (Yuan and Ye, 2013). By contrast, the present study focused on the HbO values, as they offer results that are more direct than HbR (Plichta et al., 2006).

We employed the fNIRS technique directly because of its inherent advantages in the analyses of sound-evoked activation: indeed, as a brain-imaging tool, it works non-invasive and silent. Lateralization means a more blood flow in one hemisphere in fNIRS. With this, we assume that more neuronal activity has been made in that region (but, this might be caused by different capillaries in that region or from other aspect, which deserves further neurophysiological inverstigation). We obtained all of the music stimuli results by examining the gap between the mean value and the minimum value of the HbO taken from individual channels covering the auditory cortex. This study observed a pattern of HbO activation that supports the view of functional lateralization in the auditory cortex. Since a variety of light penetrates through the scalp, we can get reliable experimental results by using the difference between the mean and the minimum value of the HbO with two criteria (*t* and *p* values) for statistical analyses. A possible explanation for the difference between S3 and S4 could be due to the influence of emotional behavior on the classical music. Music may contain features that evoke positive (or negative) emotional responses. Hemispheric lateralization increases when noise appeared in S4, which was different from S3.

Another possible explanation for the differences between the results of S3 and S4 could be the full-length music played in the background while introducing the different level of noise. *Für Elise* composed by Ludwig van Beethoven is a commonly known music that everyone can recognize and possibly imagine. Prior research has shown that music perception and music imagination has similarities in brain activation and neural mechanism (Zatorre et al., 1996). If a continuous music was cut off due to noise for a short period of time, the noise level should not make a difference if the subject can imagine the missing portion of the music. These findings further support the idea of the specialization in the auditory cortex evoked by audio stimuli.

## Conclusions

The main finding of this study was that background noise affects the hemodynamic response in the activation of the auditory cortex in music processing. Under various noise levels throughout the four different stages involving ten conditions of the experiment, the involvement of the right hemisphere's auditory cortex was higher than that of the left hemisphere (overall 73.3%, specifically, 67.1% for male and 81.1% for female). Particularly, the subjects listened to the music segments, the lateralization was 75%. Also, if only noise segments were heard, the lateralization was about 65%. But if the music was mixed with noise, such lateralization tendency became intensified. Particularly, the music + noise segments condition revealed 100% right-lateralization when the music level was 10~15% and the noise level was 10%. However, the noise level became too big (i.e., noise 20%, music 10%), the lateralization had reduced. This is an indication that too much noise diminishes the human's efforts to discern the music from surrounding noises. The obtained results support the theory that the brain is divided into compartments specializing in specific functions.

## Author contributions

Hendrik Santosa performed the experiment and carried out the data processing, Melissa Jiyoun Hong provided suggestions to improve the manuscript, and Keum-Shik Hong supervised the research and corrected the entire manuscript. All of the authors read and approved the final manuscript.

### Conflict of interest statement

The authors declare that the research was conducted in the absence of any commercial or financial relationships that could be construed as a potential conflict of interest.

## References

[B1] Aqil M. Hong K.-S. Jeong M.-Y. Ge S. S. (2012a). Cortical brain imaging by adaptive filtering of NIRS signal. Neurosci. Lett. 514, 35–41. 10.1016/j.neulet.2012.02.048 22395086

[B2] Aqil M. Hong K.-S. Jeong M.-Y. Ge S. S. (2012b). Detection of event-related hemodynamic response to neuroactivation by dynamic modeling of brain activity. Neuroimage 63, 553–568. 10.1016/j.neuroimage.2012.07.006 22796989

[B3] Bever T. G. Chiarello R. J. (1974). Cerebral dominance in musicians and nonmusicians. Science 185, 537–539. 10.1126/science.185.4150.537 4841585

[B4] Bhutta M. R. Hong K.-S. Kim B.-M. Hong M. J. Kim Y.-H. Lee S.-H. (2014). Note: three wavelengths near-infrared spectroscopy system for compensating the light absorbance by water. Rev. Sci. Instrum. 85, 026111. 10.1063/1.4865124 24593411

[B5] Boulenger V. Shtyrov Y. Pulvermuller F. (2012). When do you grasp the idea? MEG evidence for instantaneous idiom understanding. Neuroimage 59, 3502–3513. 10.1016/j.neuroimage.2011.11.011 22100772

[B6] Dehaene-Lambertz G. Dehaene S. Hertz-Pannier L. (2002). Functional neuroimaging of speech perception in infants. Science 298, 2013–2015. 10.1126/science.1077066 12471265

[B7] Friston K. J. Ashburner J. T. Kiebel S. Nichols T. Penny W. (2008). Statistical Parametric Mapping: The Analysis of Functional Brain Images. San Diego: Academic Press.

[B8] Fuchino Y. Sato H. Maki A. Yamamoto Y. Katura T. Obata A.. (2006). Effect of fMRI acoustic noise on sensorimotor activation examined using optical topography. Neuroimage 32, 771–777. 10.1016/j.neuroimaging.2006.04.197 16829140

[B9] Gaab N. Gabrieli J. D. E. Glover G. H. (2007). Assessing the influence of scanner background noise on auditory processing. II. an fMRI study comparing auditory processing in the absence and presence of recorded scanner noise using a sparse design. Hum. Brain Mapp. 28, 721–732. 10.1002/hbm.20299 17089376 PMC6871331

[B10] Giraud A. L. Kleinschmidt A. Poeppel D. Lund T. E. Frackowiak R. S. J. Laufs H. (2007). Endogenous cortical rhythms determine cerebral specialization for speech perception and production. Neuron 56, 1127–1134. 10.1016/j.neuron.2007.09.038 18093532

[B11] Guhn A. Dresler T. Andreatta M. Muller L. D. (2014). Medial prefrontal cortex stimulation modulates the processing of conditioned fear. Front. Behav. Neurosci. 8:44. 10.3389/fnbeh.2014.00044 24600362 PMC3927128

[B12] Headley D. B. Pare D. (2013). In sync: Gamma oscillations and emotional memory. Front. Behav. Neurosci. 7:170. 10.3389/fnbeh.2013.00170 24319416 PMC3836200

[B13] Hong K.-S. Nguyen H.-D. (2014). State-space of impulse hemodynamic responses over motor, somatosensory, and visual cortices. Biomed. Opt. Express 5, 1778–1798. 10.1364/BOE.5.001778 24940540 PMC4052911

[B14] Hu X.-S. Hong K.-S. Ge S. S. (2011). Recognition of stimulus-evoked neuronal optical response by identifying chaos levels of near-infrared spectroscopy time series. Neurosci. Lett. 504, 115–120. 10.1016/j.neulet.2011.09.011 21945547

[B15] Hu X.-S. Hong K.-S. Ge S. S. (2012). fNIRS-based online deception decoding. J. Neural. Eng. 9:026012. 10.1088/1741-2560/9/2/026012 22337819

[B16] Hu X.-S. Hong K.-S. Ge S. S. (2013). Reduction of trial-to-trial variability in functional near-infrared spectroscopy signals by accounting for resting-state functional connectivity. J. Biomed. Opt. 18:017003. 10.1117/1.JBO.18.1.017003 23291618

[B17] Hu X.-S. Hong K.-S. Ge S. S. Jeong M.-Y. (2010). Kalman estimator- and general linear model-based on-line brain activation mapping by near-infrared spectroscopy. Biomed. Eng. Online 9:82. 10.1186/1475-925X-9-82 21138595 PMC3020171

[B18] Jancke L. Mirzazade S. Shah N. J. (1999). Attention modulates activity in the primary and the secondary auditory cortex: a functional magnetic resonance imaging study in human subjects. Neurosci. Lett. 266, 125–128. 10.1016/S0304-3940(99)00288-8 10353343

[B19] Jancke L. Wustenberg T. Scheich H. Heinze H. J. (2002). Phonetic perception and the temporal cortex. Neuroimage 15, 733–746. 10.1006/nimg.2001.1027 11906217

[B20] Kamran M. A. Hong K.-S. (2013). Linear parameter-varying model and adaptive filtering technique for detecting neuronal activities: an fNIRS study. J. Neural. Eng. 10:056002. 10.1088/1741-2560/10/5/056002 23893789

[B21] Kamran M. A. Hong K.-S. (2014). Reduction of physiological effects in fNRS waveforms for efficient brain-state decoding. Neurosci. Lett. 580, 130–136. 10.1016/j.neulet.2014.07.058 25111978

[B22] Katsunuma A. Takamori H. Sakakura Y. Hamamura Y. Ogo Y. Katayama R. (2002). Quiet MRI with novel acoustic noise reduction. Magn. Reson. Mater. Phys. 13, 139–144. 10.1016/S1352-8661(01)00142-9 11755088

[B23] Khan M. J. Hong M. J. Hong K.-S. (2014). Decoding of four movement directions using hybrid NIRS-EEG brain-computer interface. Front. Hum. Neurosci. 8:244. 10.3389/fnhum.2014.00244 24808844 PMC4009438

[B24] Kovelman I. Shalinsky M. H. White K. S. Schmitt S. N. Berens M. S. Paymer N.. (2009). Dual language use in sign-speech bimodal bilinguals: fNIRS brain-imaging evidence. Brain. Lang. 109, 112–123. 10.1016/j.bandl.2008.09.008 18976807 PMC2749876

[B25] Kraus N. Chandrasekaran B. (2010). Music training for the development of auditory skills. Nat. Rev. Neurosci. 11, 599–605. 10.1038/nrn2882 20648064

[B26] Kuhnis J. Elmer S. Jancke L. (2014). Auditory evoked responses in musicians during passive vowel listening are modulated by functional connectivity between bilateral auditory-related brain regions. J. Cogn. Neurosci. 26, 2750–2761. 10.1162/jocn_a_00674 24893742

[B27] Kuhnis J. Elmer S. Meyer M. Jancke L. (2013a). Musicianship boosts perceptual learning of pseudoword-chimeras: an electrophysiological approach. Brain Topogr. 26, 110–125. 10.1007/s10548-012-0237-y 22736323

[B28] Kuhnis J. Elmer S. Meyer M. Jancke L. (2013b). The encoding of vowels and temporal speech cues in the auditory cortex of professional musicians: an EEG study. Neuropsychologia 51, 1608–1618. 10.1016/j.neuropsychologia.2013.04.007 23664833

[B29] Mansfield P. Haywood B. Coxon R. (2001). Active acoustic control in gradient coils for MRI. Magn. Reson. Med. 46, 807–818. 10.1002/mrm.1261 11590659

[B30] May A. C. Stewart J. L. Tapert S. E. Paulus M. P. (2014). The effect of age on neural processing of pleasant soft touch stimuli. Front. Behav. Neurosci. 8:52. 10.3389/fnbeh.2014.00052 24600366 PMC3930859

[B31] McJury M. Stewart R. W. Crawford D. Toma E. (1997). The use of active noise control (ANC) to reduce acoustic noise generated during MRI scanning: some initial results. Magn. Reson. Med. 15, 319–322. 10.1016/S0730-725X(96)00337-2 9201679

[B32] Meyer M. Baumann S. Jancke L. (2006). Electrical brain imaging reveals spatio-temporal dynamics of timbre perception in humans. Neuroimage 32, 1510–1523. 10.1016/j.neuroimage.2006.04.193 16798014

[B33] Naseer N. Hong K.-S. (2013). Classification of functional near-infrared spectroscopy signals corresponding to the right- and left-wrist motor imagery for development of a brain-computer interface. Neurosci. Lett. 553, 84–89. 10.1016/j.neulet.2013.08.021 23973334

[B34] Naseer N. Hong M. J. Hong K.-S. (2013). Online binary decision decoding using functional near-infrared spectroscopy for the development of brain-computer interface. Exp. Brain Res. 232, 555–564. 10.1007/s00221-013-3764-1 24258529

[B35] Ono K. Nakamura A. Yoshiyama K. Kinkori T. Bundo M. Kato Y.. (2011). The effect of musical experience on hemispheric lateralization in musical feature processing. Neurosci. Lett. 496, 1414–1145. 10.1016/j.neulet.2011.04.002 21513771

[B36] Perani D. Saccuman M. C. Scifo P. Spada D. Andreolli G. Rovelli R.. (2010). Functional specializations for music processing in the human newborn brain. Proc. Natl. Acad. Sci.U.S.A. 107, 4758–4763. 10.1073/pnas.0909074107 20176953 PMC2842045

[B37] Plichta M. M. Herrmann M. J. Baehne C. G. Ehlis A. C. Richter M. M. Pauli P.. (2006). Event-related functional near-infrared spectroscopy (fNIRS): are the measurements reliable? Neuroimage 31, 116–124. 10.1016/j.neuroimage.2005.12.008 16446104

[B38] Santosa H. Hong M. J. Kim S.-P. Hong K.-S. (2013). Noise reduction in functional near-infrared spectroscopy signals by independent component analysis. Rev. Sci. Instrum. 84, 073106. 10.1063/1.4812785 23902043

[B39] Scarff C. J. Dort J. C. Eggermont J. J. Goodyear B. G. (2004). The effect of MR scanner noise on auditory cortex activity using fMRI. Hum. Brain Mapp. 22, 341–349. 10.1002/hbm.20043 15202112 PMC6871718

[B40] Schuppert M. Münte T. F. Wieringa B. M. Altenmüller E. (2000). Receptive amusia: evidence for cross-hemispheric neural networks underlying music processing strategies. Brain 123, 546–559. 10.1093/brain/123.3.546 10686177

[B41] Shirao N. Okamoto Y. Mantani T. Okamoto Y. Yamawaki S. (2005). Gender differences in brain activity generated by unpleasant word stimuli concerning body image: an fMRI study. Br. J. Psychiatry 186, 48–53. 10.1192/bjp.186.1.48 15630123

[B42] Shtyrov Y. Kujala T. Ahveninen J. Tervaniemi M. Alku P. Ilmoniemi R. J.. (1998). Background acoustic noise and the hemispheric lateralization of speech processing in the human brain: magnetic mismatch negativity study. Neurosci. Lett. 251, 141–144. 10.1016/S0304-3940(98)00529-1 9718994

[B43] Shtyrov Y. Smith M. L. Horner A. J. Henson R. Nathan P. J. Bullmore E. T.. (2012). Attention to language: novel MEG paradigm for registering involuntary language processing in the brain. Neuropsychologia 50, 2605–2616. 10.1016/j.neuropsychologia.2012.07.012 22820635 PMC3657698

[B44] Sinai A. Pratt H. (2003). High-resolution time course of hemispheric dominance revealed by low-resolution electromagnetic tomography. Clin. Neurophysiol. 114, 1181–1188. 10.1016/S388-2457(03)000087-7 12842713

[B45] Soekadar S. R. Witkowski M. Cossio E. G. Birbaumer N. Cohen L. G. (2014). Learned EEG-based brain self-regulation of motor-related oscillations during application of transcranial electric brain stimulation: feasibility limitations. Front. Behav. Neurosci. 8:93. 10.3389/fnbeh.2014.00093 24672456 PMC3957028

[B46] Tervaniemi M. Hugdahl K. (2003). Lateralization of auditory-cortex functions. Brain Res. Rev. 43, 231–246. 10.1016/j.brainresrev.2003.08.004 14629926

[B47] Tervaniemi M. Medvedev S. V. Alho K. Pakhomov S. V. Roudas M. S. van Zuijen T. L.. (2000). Lateralized automatic auditory processing of phonetic versus musical information: a PET study. Hum. Brain. Mapp. 10, 74–79. 10.1002/(SICI)1097-0193(200006)10:2<74::AID-HBM30>3.0.CO;2-2 10864231 PMC6872017

[B48] Toga A. W. Thompson P. M. (2003). Mapping brain asymmetry. Nat. Rev. Neurosci. 4, 37–48. 10.1038/nrn1009 12511860

[B49] Turnip A. Hong K.-S. (2012). Classifying mental activities from EEG-P300 signals using adaptive neural networks. Int. J. Innov. Comput. Inform. Control 8, 6429–6443.

[B50] Turnip A. Hong K.-S. Jeong M.-Y. (2011). Real-time feature extraction of P300 component using adaptive nonlinear principal component analysis. Biomed. Eng. Online 10:83. 10.1186/1475-925X-10-83 21939560 PMC3749271

[B51] Warrier C. Wong P. C. M. Penhune V. Zatorre R. Parrish T. Abrams D.. (2009). Relating structure to function: Heschl's gyrus and acoustic processing. J. Neurosci. 29, 61–69. 10.1523/JNEUROSCI.3489-08.2009 19129385 PMC3341414

[B52] Wong P. C. M. Ettlinger M. Sheppard J. P. Gunasekera G. M. Dhar S. (2010). Neuroanatomical characteristics and speech perception in noise in older adults. Ear. Hear. 31, 471–479. 10.1097/AUD.0b013e3181d709c2 20588117 PMC2919052

[B53] Wong P. C. M. Uppunda A. K. Parrish T. B. Dhar S. (2008). Cortical mechanisms of speech perception in noise. J. Speech Lang. Hear. R. 51, 1026–1041. 10.1044/1092-4388(2008/075) 18658069

[B54] Ye J.-C. Tak S. Jang K.-E. Jung J. Jang J. (2009). NIRS-SPM: statistical parametric mapping for near-infrared spectroscopy. Neuroimage 44, 428–447. 10.1016/j.neuroimage.2008.08.036 18848897

[B55] Yuan Z. Ye J.-C. (2013). Fusion of fNIRS and fMRI data: identifying when and where hemodynamic signals are changing in human brains. Front. Hum. Neurosci. 7:676. 10.3389/fnhum.2013.00676 24137124 PMC3797402

[B56] Zaehle T. Jancke L. Meyer M. (2007). Electrical brain imaging evidences left auditory cortex involvement in speech and non-speech discrimination based on temporal features. Behav. Brain Funct. 3:63. 10.1186/1744-9081-3-63 18070338 PMC2231369

[B57] Zatorre R. J. Halpern A. R. Perry D. W. Meyer E. Evans A. C. (1996). Hearing in the mind's ear: a PET investigation of musical imagery and perception. J. Cogn. Neurosci. 8, 29–46. 10.1162/jocn.1996.8.1.29 23972234

[B58] Zhou Y. Li S. Dunn J. Li H. D. Qin W. Zhu M. H.. (2014). The neural correlates of risk propensity in males and females using resting-state fMRI. Front. Behav. Neurosci. 8:2. 10.3389/fnbeh.2014.00002 24478649 PMC3904110

